# Liver fibrosis progression in a cohort of young HIV and HIV/ HBV co-infected patients: A longitudinal study using non-invasive APRI and Fib-4 scores

**DOI:** 10.3389/fmed.2022.888050

**Published:** 2022-07-29

**Authors:** Diana Gabriela Iacob, Monica Luminos, Otilia Elisabeta Benea, Ana-Maria Tudor, Cristina Mihaela Olariu, Simona Alexandra Iacob, Simona Ruta

**Affiliations:** ^1^Department of Infectious Diseases, Emergency University Hospital, Bucharest, Romania; ^2^Department of Infectious Diseases and Virology, Carol Davila University of Medicine and Pharmacy, Bucharest, Romania; ^3^Department of Infectious Diseases, National Institute of Infectious Diseases “Prof. Dr. Matei Bals”, Bucharest, Romania; ^4^Department of Emerging Viral Infections and HIV/AIDS International Research Center, Stefan S. Nicolau Institute of Virology, Bucharest, Romania

**Keywords:** liver fibrosis, APRI score, Fib-4 score, HIV, HIV/HBV co-infection, non-invasive score, antiretroviral therapy, longitudinal assessment

## Abstract

**Background:**

The risk of liver fibrosis increases over time in HIV and HIV-HBV individuals even under antiretroviral treatment (ART), warranting a rigorous and periodic monitorization. Given the lower availability of transient elastography, we aimed to assess the longitudinal variation of two non-invasive liver fibrosis scores, APRI and Fib-4, in cases with HIV monoinfection, HIV-HBV co-infection and individuals with HBsAg-seroclearance.

**Methods:**

We performed an observational retrospective study between 2013 and 2019 on 212 HIV patients including 111 individuals with HIV mono-infection, 62 individuals with HIV-HBV co-infection and positive HBsAg and 39 cases with HIV-HBV infection and HBsAg-loss. The groups were followed at 36, 48, and 60 months. Liver fibrosis was indicated by an APRI >0.5 or Fib-4≥1.45 score and advanced fibrosis by an APRI score >1.5 or Fib-4 >3.25. Logistic regression with generalized estimating equations (GEE) was used to assess the predictors for the presence of liver fibrosis over time.

**Results:**

During a median follow-up of 58.5 months the prevalence of liver fibrosis in all patients increased with 0.5% reaching 11.3% using an APRI score and with 0.9% reaching 10.8% using the Fib-4 score. At the visit corresponding to 60 months the prevalence of liver fibrosis was higher in all HIV-HBV patients compared with individuals with HIV mono-infection, namely: 16.1% on APRI and 12.9% on the Fib-4 score in HIV-HBV/HBsAg-positive individuals, 12.8% on both APRI and Fib-4 scores in HIV-HBV/HBsAg-negative individuals vs. 8.1 and 9%, respectively in HIV mono-infection. The presence of liver fibrosis over the study period was independently associated with plasma HIV RNA, CD4+T cell counts, HIV-HBV co-infection (for APRI >0.5) and ART non-adherence (for Fib-4 >1.45). At the final visit, non-adherence to ART and CD4+T cell counts remained associated with liver fibrosis.

**Conclusions:**

The study found a slow progression of APRI and Fib-4 scores over time in young PLWH with extensive ART. Liver fibrosis scores continued to increase in patients with HIV mono-infection yet remained lower than in HIV-HBV patients irrespective on the presence of HBsAg. The periodic follow-up using non-invasive scores on the long-term could help improve the surveillance in low-income settings and high scores should be followed by additional diagnostic methods.

## Introduction

Liver disease is one of the most important causes of mortality and morbidity in people living with HIV (PLWH) due to its progression to liver fibrosis and cirrhosis, as well as to the development of hepatocellular carcinoma. Overall, liver-related deaths account for 13–15% of fatalities in PLWH and liver disease remains one of the main causes of death not related to the acquired immunodeficiency syndrome (AIDS) (1, 2).

The prevalence of liver fibrosis in PLWH with HIV monoinfection reaches 11–17% of patients, while the prevalence of fibrosis in patients co-infected with hepatitis viruses varies between 12 and 43% (3–5). The pathogenesis of liver fibrosis in PLWH involves multiple mechanisms including direct effects of HIV replication, persistent immune activation, metabolic syndrome, cumulative toxicity due to antiretroviral treatment (ART), alcohol and drug abuse as well as viral co-infections (6). Hepatitis B virus (HBV) is one of the most frequent HIV co-infections, affecting an estimated 2.7 million people (7.6% of PLWH). HBV co-infection aggravates the course of liver disease and favors the progression to cirrhosis and the development of hepatocellular carcinoma. Following ART, the rate of HBsAg loss in HIV-HBV co-infected individuals has been shown to be higher compared with treated HBV monoinfection (7–9). Nevertheless, the HBV infection has been shown to be associated with a higer mortality in PLWH irrespective on the presence active or previous HBV replication and cases which have already developed moderate or advanced liver fibrosis are less likely to display a histologic regression of the liver injury after HBeAg or HBsAg seroclearence (10, 11). Additionally, even after HBsAg loss, these patients are advised to continue the administration of antiretroviral agents (ARV) with dual HIV and HBV activity in order to prevent viral HBV reactivation (12, 13). In this context, current recommendations on the surveillance of liver disease include a periodic follow-up of liver fibrosis in all cases with HIV-HBV co-infection, while individuals with HIV monoinfection are monitored using liver transaminases at 3–12 months (14, 15).

The initiation of ART has been shown to attenuate the progression of liver fibrosis in both HIV and HIV-HBV co-infected patients (16, 17), yet a certain subset of patients in both groups can continue to display a progression of liver fibrosis despite long-term ART (18–21). While most studies on patients with HIV on prolonged treatment include middle-aged patients (11, 22) the detection of liver fibrosis has also been documented in children vertically infected with HIV. Thus, studies using the non-invasive APRI score indicated that the prevalence of liver fibrosis (APRI >0.5) in children with HIV varies between 6.5 and 10%, while the prevalence of advanced fibrosis (APRI >1.5) ranges between 0.8 and 3.2% (20, 23, 24). Furthermore, a study by Kapogiannis on children with HIV monoinfection on ART showed that APRI and Fib-4 scores increase annualy with 2 and 6%, respectively (20). On the other hand, young HIV-HBV co-infected patients in their early twenties and thirties on long-term ART have been less studied. Data on this particular population shows a higher prevalence of liver fibrosis compared with HIV monoinfection and a large variability between 6.7 and 33% (18, 25) even in the absence of other risk factors. Hence, the study of liver fibrosis in young HIV and HIV-HBV adults with prolonged ART could offer a key understanding on the development of liver complications in these patient groups even in the absence of other risk factors associated with liver disease.

The current study aims to investigate the prevalence of liver fibrosis in PLWH with extensive ART and includes patients infected with HIV during childhood beginning in the early 1990's. It is estimated that Romania houses over 5,500 PLWH, who have acquired HIV infection horizontally in early childhood, due to unsafe parenteral methods and who are currently in their twenties and thirties (26, 27). Approximately 44% of these PLWH have been diagnosed with HBV co-infection during the same time frame. Nevertheless, data on the liver morbidity and prevalence of liver fibrosis in this young group of Romanian patients with prolonged ART exposure is scarce (28, 29). Additionally, no data is available yet on the longitudinal changes in the prevalence of liver fibrosis in these young Romanian patients with HBV coinfection, after HBsAg loss.

The current study aims to assess the prevalence of liver fibrosis using two non-invasive liver fibrosis scores, APRI and Fib-4, in a cohort of individuals with HIV monoinfection and HIV-HBV infection on long term ART including a specific group of HBV individuals with HBsAg loss and a group with seroconversion to HBsAb (most probably due to a functional cure on ART).

The study employed data recorded at annual visits performed at 36, 48, and 60 months, respectively. The study also analyzes the predictors for liver fibrosis over time and separately, the risk factors which remained associated with liver fibrosis at the last visit.

## Materials and methods

### Study population

We performed a retrospective observational study on 212 PLWH followed at “Prof. dr. Matei Bals” Institute of Infectious Diseases, in Bucharest Romania. The study included 111 patients with HIV mono-infection, 62 patients with HIV-HBV co-infection and a third group of 39 individuals with HIV-HBV co-infection and negative HBsAg. The third group thus included patients with a previous record of chronic HBV hepatitis, who had cleared the HBsAg and currently displayed positive HBcIgG Ab and negative HBsAg, with or without anti-HBs Ab. For clarity, this group also included three patients who developed anti-HBs Ab during the follow-up period.

The eligibility criteria included patients between 18 and 65 years old diagnosed with HIV. Patients were excluded if they displayed positive serologic assays for hepatitis A, C, D, or E, a significant alcohol consumption >2 drinks/day, hepatocellular carcinoma or other AIDS or non-AIDS related neoplasms. All patients were receiving ART with nucleoside/nucleotide analog reverse-transcriptase inhibitors (NRTIs) including two nucleot(s)ide analogs active against HBV, namely lamivudine (3TC) and tenofovir disoproxil fumarate (TDF) or tenofovir alafenamide (TAF). Of note, all patients had started ART at least 1 year before the baseline assessment.

### Methods

Clinical data included age, gender, date of HIV diagnosis, date of ART initiation, ART regimens throughout the follow-up period and adherence to treatment, as well as the presence of hypertension, diabetes, and obesity according to medical records. BMI measurements were available only on the last assessment.

The diagnosis of chronic HBV co-infection was based on the presence of HBsAg at two separate assessments 6 months apart.

Considering the WHO definition of adherence as “the extent to which a person's behavior—taking medication, following a diet and/or executing lifestyle changes, corresponds with agreed recommendations from a health care provider” (30), the adherence to treatment was quantified using the monthly released pills. Hence, patients who failed to present for ART were considered non-adherent. The level of adherence to ART was divided into an optimal adherence ≥95%, a good adherence between 80 and 95%, moderate adherence 60–80% and low adherence <60%.

Laboratory data included complete blood count, fibrinogen, erythrocyte sedimentation prevalence (ESR), C reactive protein, AST, ALT, alkaline phosphatase, gamma-glutamyl transpeptidase (GGT), total proteins, albumin and alpha-fetoprotein, blood glucose, cholesterol, HDL-Cholesterol, triglyceride levels, CD4+ T cell counts, plasma HIV RNA, HBsAg, anti-HBs Ab, anti-HBe Ab, plasma HBV DNA. AST and ALT upper limit of normal was determined by the laboratory, in accordance with the assay protocol.

The APRI score was calculated using formula [(AST/upper normal limit of AST)] x 100 / Platelet Count), while the Fib-4 score was calculated as [age (years) x AST (U/L)] / [platelet count (10^9^/L) x ALT^1/2^ (U/L)].

The presence of liver fibrosis was defined using two separate end points APRI>0.5 and Fib-4 ≥1.45. Advanced liver fibrosis was defined using either an APRI >1.5 or a Fib-4 score >3.25. The values of APRI and Fib-4 scores were presented along with the proportion of patients who displayed liver fibrosis based on the threshold of 0.5 for APRI and 1.45 for the Fib-4 score.

Laboratory data and liver fibrosis scores were retrieved retrospectively between 2013 and 2019 and included the periodic visits planned over a 5-year period (60 months). The baseline was considered as the assessment performed 5 years prior. Subsequently, the analysis included the visits corresponding to the assessment at 36, 48, and 60 months. In cases with a later presentation, the first presentation after 60 months was used.

Regarding the interval for the collection of data, the median follow-up for the second visit was of 34.4 months (IQR = 30, 38), while the median follow-up for the third visit was of 46.9 months (IQR = 42.4, 51).

At the last visit, the median duration of follow-up was of 58.5 months (IQR = 54, 63) in all patients, namely of 59 months (IQR = 54, 64) in patients with HIV mono-infection and 58 months (IQR = 54, 61) months in patients with HIV-HBV co-infection.

All patients provided written informed consent and the protocol was approved by the Ethics Committee in accordance with the Helsinki Declaration.

### Statistical analysis

Categorical variables were expressed as absolute frequencies and percentages and the differences between these were assessed using chi-square tests and Fisher exact tests. Continuous variables were expressed using mediands and interquartile range in accordance with the results of normality tests and the differences between these variables were comparated using Mann Whitney U, Kruskal Wallis test and Wilcoxon Rank Sum test, respectively for related samples.

We analyzed the predictors associated with the presence of liver fibrosis (as assessed with an APRI score >0.5 or with a Fib-4 score >1.45) using a generalized estimating equations (GEE) logistic model. An independent correlation structure was selected based on the model fit measured by the quasi-likelihood under the independence model criterion.

A GEE model was chosen to account for the correlations between repeated measurements. In the longitudinal analysis with 848 cases (212 patients with four subsequent measurements) an APRI >0.5 was found in 93 cases vs. 718 cases (37 missing cases), while a Fib-4 score ≥1.45 was found in 83 cases vs. 727 cases (38 missing cases).

A *p*-value < 0.05 was considered significant. *Post-hoc* tests with Bonferroni corrections were applied to adjust for multiple group comparisons.

Statistical analysis was performed using IBM SPSS Statistics for Windows, version 27.0.1.0 (IBM Corp., Armonk, N.Y., USA).

## Results

### Clinical and laboratory assessment of the study population

The study followed 212 PLWH on long-term ART for a median of 58.5 months.

At inclusion, the median age in all patients was of 30 years and 121 (57.1%) of all patients were below 35 years of age. The median HIV duration at baseline was of 13 years (IQR = 8, 14) and the median ART duration was of 10.5 years (IQR = 5, 13). The characteristics of each group of patients at baseline and at the last visit are represented in Tables 1, 2.

**Table 1:** Patient characteristics and laboratory data at baseline in individuals with HIV mono-infection, HIV-HBV co-infection with positive HBsAg and individuals with HIV-HBV co-infection and HBsAg loss.

**Patient characteristics and laboratory data**	**HIV group** **(*****N*** = **111)**	**HIV-HBV group** **(*****N*** = **62)**	**HIV-HBV with** **HBsAg loss** **(*****N*** = **39)**	***P*** **-value**
Male Gender (%)	57 (51.4%)	36 (58%)	21 (53.8%)	0.697
Median Age, IQR (years)	35 (26, 45)	26 (25, 31)	34 (25, 43)	<0.001
Median HIV duration, IQR (years)	12 (6, 14)	14 (11, 14)	9 (6, 13)	0.003
Median ART duration, IQR (years)	9 (4, 13)	13 (7, 13)	5.5 (4.75, 8.5)	0.093
Median Lymphocytes, IQR (cells/μL)	2,060 (1490, 2540)	1,840 (1400, 2445)	1,740 (1427, 1950)	0.668
Median Thrombocytes, IQR (cells/μL)	2,61,500 (2,22,750, 3,0,000)	2,13,000 (1,78,000, 2,65,750)	2,52,000 (1,86,500, 2,71,250)	<0.001
Median INR, IQR	0.97 (0.92, 1.04)	0.98 (0.95, 1.05)	0.97 (0.94, 1)	0.447
Median AST, IQR (IU/L)	24 (19, 30)	40 (29, 51)	25 (19, 32)	<0.001
AST > ULN^*^, (%)	6 (5.4%)	8 (12.9%)	1 (2.6%)	0.081
Median ALT, IQR (IU/L)	28 (21, 36.25)	51.5 (31, 66)	29 (23, 36)	<0.001
ALT > ULN^*^, (%)	3 (2.7%)	14 (22.6%)	0	<0.001
Median ALP, IQR (IU/L)	75 (60, 98)	73 (62, 91)	64.5 (58, 85.5)	0.305
Median GGT, IQR (IU/L)	33 (17, 57)	33 (20.5, 58)	21 (14, 47.5)	0.319
Total bilirubin >2 mg/dl (%)	7 (6.3%)	1 (1.6%)	3 (7.69%)	0.116
Median serum glucose, IQR (mg/dl)	95 (86, 102)	90 (84, 97)	92 (87, 97)	0.148
Median serum Cholesterol, IQR (mg/dl)	208 (170, 256)	181 (152, 209)	196 (155, 226)	0.008
Cholesterol >200 mg/dl (%)	54 (48.6%)	17 (27.41%)	14 (35.89%)	0.024
Median tryglicerides, IQR (mg/dl)	155 (96.25, 273.25)	133 (98, 228)	146 (83, 267)	0.519
Tryglicerides >150 mg/dl (%)	51 (45.9%)	22 (35.5%)	13 (33.3%)	0.389
Median HDL-Cholesterol, IQR (mg/dl)	47.8 (38.9, 57.8)	44.4 (39.4, 61.4)	45 (38, 57.2)	0.963
HDL- Cholesterol <0 mg/dl (%)	28 (25.2%)	14 (22.6%)	8 (20.51%)	0.837
Median serum proteins, IQR (%)	7.9 (7.4, 8.1)	7.8 (7.5, 8.3)	7.8 (7.8, -)	0.727
Median albumin, IQR (mg/dl)	4.45 (4.66, 4.94)	4.56 (4.39, 4.87)	5 (4.66, 5)	0.016
Median AFP, IQR (ng/ml)	2.3 (1.6, 2.9)	2.3 (1.8, 4.6)	1.7 (1.1, -)	0.738
Median plasma log10 HIV RNA, IQR (copies/ml)	2.22 (1.31, 4.09)	2.36 (1.3, 3.78)	1.84 (1.3, 3.14)	0.697
Detectable plasma HIV RNA (%)	47 (42.3%)	31 (50%)	17 (43.58%)	0.663
Median CD4 T cell count, IQR (cells/mm^3^)	531 (238, 794)	369 (270, 693)	524 (270, 726)	0.852
CD4 T cell count <200 cells/mm^3^ (%)	13 (11.7%)	5 (8.1%)	2 (5.12%)	0.388
CD4 T cell count <500 cells/mm^3^ (%)	40 (36%)	25 (40.3%)	11 (28.2%)	0.355

*Continuous variables are represented using medians (IQR), while categorical variables are displayed using the number (%) of cases*.

* *ULN, upper limit of normal (according to the laboratory kit and producer recommendations)*.

**Table 2:** Laboratory characteristics and comparisons corresponding to the visit corresponding to 60 months in individuals with HIV, HIV-HBV co-infection with positive HBsAg and individuals with HIV-HBV and HBsAg loss.

**Laboratory characteristics**	**HIV group** **(*****N*** = **111)**	**HIV-HBV group** **(*****N*** = **62)**	**HIV-HBV with** **HBsAg loss** **(*****N*** = **39)**	***P*** **-value**
Median Lymphocytes, IQR (cells/μL)	2,220 (1,900, 2,797)	1,935 (1,540, 2,545)	2,140 (1,820, 2,760)	0.060
Median Thrombocytes, IQR (cells/μL)	2,48,500 (1,95,250, 2,73,500)	2,14,000 (1,53,250, 2,72,000)	2,62,000 (1,99,000, 3,01,000)	0.016
Median INR, IQR (IU/L)	0.97 (0.92, 1.04)	0.98 (0.95, 1.04)	0.99 (0.96, 1.04)	0.363
Median AST, IQR (IU/L)	26 (20, 34)	33 (25, 41.5)	28 (23, 36)	0.061
AST > ULN^*^ (%)	10 (9%)	8 (12.9%)	4 (10.2%)	0.710
Median ALT, IQR (IU/L)	29 (21, 39)	36 (24, 53)	33 (23, 40)	0.322
ALT > ULN^*^ (%)	16 (14.4%)	7 (11.3%)	2 (5.1%)	0.238
Median ALP, IQR (IU/L)	72 (57, 89)	71 (52, 94)	63.5 (54, 87)	0.614
Median GGT, IQR (IU/L)	32 (20, 51)	29 (22, 39)	24 (16, 33)	0.316
Total bilirubin >2 mg/dl (%)	4 (3.6%)	2 (3.2%)	1 (2.5%)	0.971
Median serum glucose, IQR (mg/dl)	94 (87, 104)	92 (87, 99)	91 (80, 106)	0.210
Serum cholesterol >200 mg/dl (%)	52 (46.8%)	19 (30.64%)	12 (30.76%)	0.262
Median serum cholesterol, IQR (mg/dl)	204 (179, 235)	192 (175, 218)	183 (148, 214)	0.038
Tryglicerides >150 mg/dl (%)	45 (40.5%)	17 (27.42%)	11 (28.20%)	0.473
Median tryglicerides, IQR (mg/dl)	141 (98, 211)	125 (90, 193)	130 (82, 230)	0.554
HDL-Cholesterol <50 mg/dl (%)	18 (16.2%)	16 (25.8%)	10 (25.64%)	0.652
Median HDL-Cholesterol, IQR (mg/dl)	48 (37, 55)	41 (33, 57)	43 (38, 57)	0.513
Median serum proteins, IQR (g/dl)	7.75 (7.4, 8.3)	7.85 (7.4, 8.2)	7.6 (7.3, 8.05)	0.625
Median Albumin, IQR (g/dl)	4.82 (4.5, 5.2)	4.96 (4.59, 5.4)	4.8 (4.3, 5.2)	0.318
Median AFP, IQR (ng/ml)	0.8 (0.17, 3.95)	1.2 (0.5, 4.1)	0.4 (0.2, 0.4)	0.339
Detectable plasma HIV RNA (%)	39 (35.1%)	22 (42.3%)	8 (20.51%)	0.107
Median plasma log10 HIV RNA, IQR (copies/ml)	1.47 (1.3, 1.72)	1.63 (1.32, 3.06)	1.7 (1.3, 2.45)	0.160
CD4 T cells <200 cells/mm^3^ (%)	1 (0.9%)	3(4.83%)	1 (2.56%)	0.269
CD4 T cells <500 cells/mm^3^ (%)	23 (20.72%)	19 (30.64%)	10 (25.64%)	0.308
Median CD4 T cell count, IQR (cells/mm^3^)	732 (521, 927)	596 (450, 827)	641 (429, 810)	0.036

*Continuous variables are represented using the median and IQR, while categorical variables are displayed using the number (%) of cases*.

* *ULN – upper limit of normal (according to the laboratory kit and producer recommendations)*.

Non-AIDS related comorbidities found in this patient cohort included compensated type 2 diabetes, affecting 11 (5.18%) patients, hypertension in 11 cases (5.18%). At the last assessment a BMI above 30 kg/m^2^ was found in 8 patients (3.77%), namely five people with HIV mono-infection, two cases with HIV-HBV co-infection and 1 case with HBsAg loss.

All patients received an ART regimen using HBV active NRTIs (either 3TC or TDF/TAF) and either a protease inhibitor (PI) or an integrase inhibitor (INSTI).

Individual data on NRTI usage revealed a wide usage of 3TC at baseline, covering 82.1% of all individuals as follows: 90.1% of patients with HIV monoinfection, 67.7% of the cases with HIV-HBV co-infection and 82.1% of PLWH who had cleared the HBsAg.

After a median of 58.5 months the administration of 3TC decreased in favor of TAF/TDF, covering 60.4% of all individuals. Thus, at the last visit 3TC was administered to 70.3% of individuals with HIV mono-infection, 45.2% of those with HIV-HBV co-infection and 56.4% of PLWH who had cleared the HBV infection.

The administration of PIs decreased over time, while the uptake of INSTIs gradually increased from 14.2% of patients at baseline to 61.3% of all cases at the last visit. Considering each group individually, the use of INSTIs increased from 13.4 to 67.6% in people with HIV mono-infection, from 21 to 54.5% of cases with HIV-HBV co-infection and from 5.1 to 51.3% of cases with previous HBV infection.

Regarding the duration of ARV exposure, most individuals showed prolonged exposure to ARV and the median ART duration at baseline was of 13 years. Furthermore, 57.1% of all individuals had received at least three previous ARV regimens at baseline, of which 50.5% individuals with HIV mono-infection, 61.3% of individuals with HIV-HBV co-infection, and 69.2% of cases with HBsAg seroclearance.

The adherence to treatment over the study period corresponded to an overall moderate adherence. Thus, according to the number of monthly pills released to these patients from baseline to the last visit, 75.2% of the patients presents for the monthly retrieval of the medication.

The results obtained for each group indicate that 82% of the cases with HIV monoinfection showed a good adherence to ART, while the adherence in cases with HIV-HBV co-infection and HBsAg loss was moderate to low. Thus, 75.8% of the individuals with HIV-HBV co-infection and 59% of the cases with HBsAg loss presented for the monthly pill-release.

Data on all patients as well as comparisons between the three studied groups at baseline and at the last visit can be consulted in Tables 1, 2.

No significant differences were observed between cases with HIV-HBV co-infection including cases with HBsAg loss regarding HIV RNA viral load or CD4 T cell counts at baseline or at the last visit.

Regarding the serologic profile of HBV in individuals with HIV-HBV co-infection, over half of the cases developed anti-HBe Ab. Thus, at baseline 37 (56.92%) people with HIV-HBV co-infection displayed anti-HBe Ab, while on the last visit the number of cases with HBe seroconversion increased to 43 (66.15%) cases.

HBV DNA was available in only 21 patients at baseline, of which 14 (66.7%) with a detectable viral load and a median value of 418 IU/ml. At the last visit, HBV DNA was available in 42 patients, of which 23 (54.8%) showed a low detectable viral load and a median value of 223 IU/ml.

### The prevalence of liver fibrosis using APRI and Fib-4 scores

**Figure 1:**
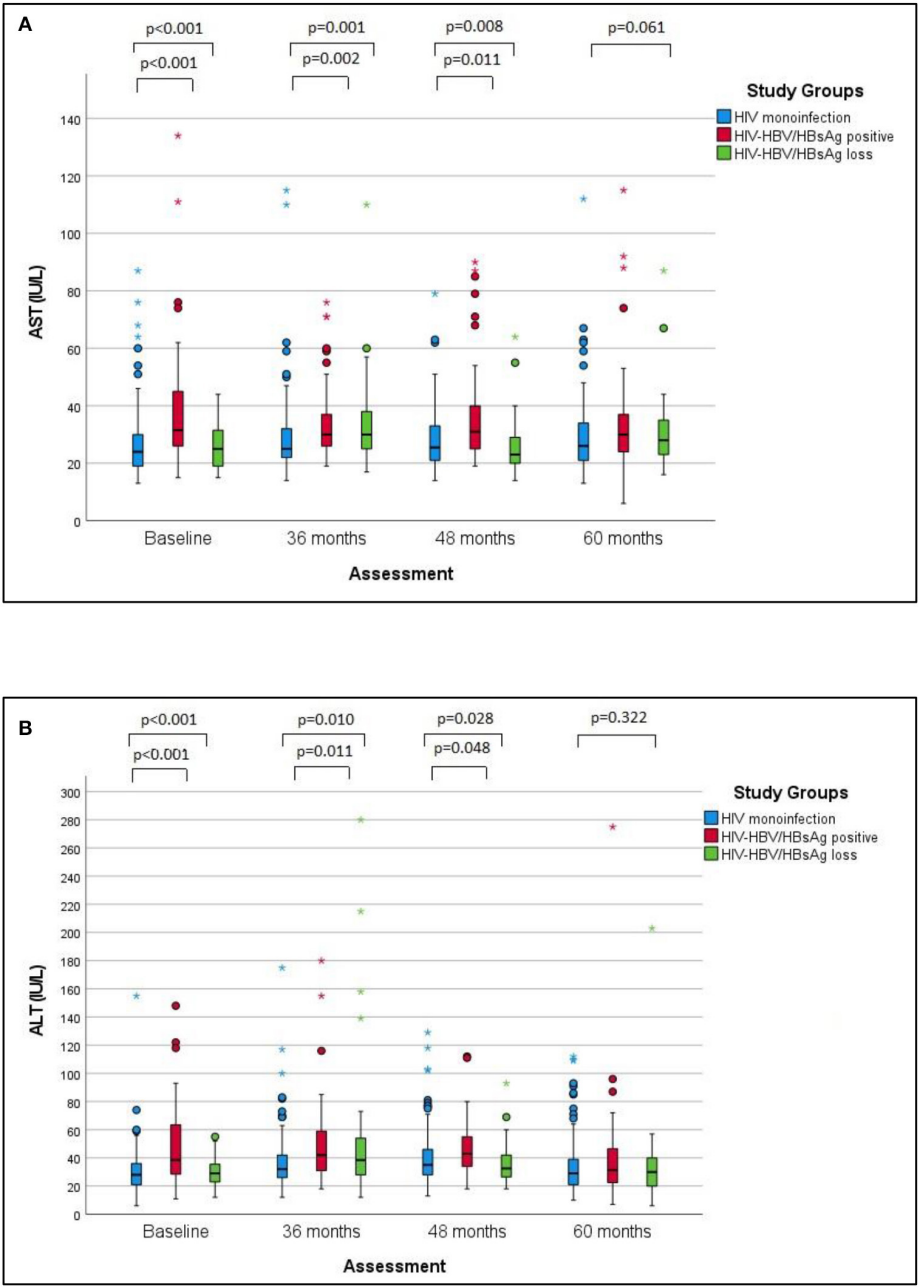
AST and ALT values over time in the studied groups. **(A)** The changes of AST values over the follow-up period in the studied groups. **(B)** The changes of ALT values over the studied period in the studied groups. The differences between groups regarding the AST and ALT values respectively decreased at baseline, 36 and 48 months, with no significant difference at 60 months. Pairwise comparisons revealed significantly higher median AST values and ALT values in cases with HIV-HBV co-infection/HBsAg versus with HIV monoinfection at baseline, 36 and 48 months.

Approximately 90% of the patients in the study population showed normal liver enzymes at baseline as well as at the last visit, with discrete fluctuations over time. In this respect, the proportion of all patients with AST values above the upper limit of normal (ULN) exhibited a discrete increase from 7.1% at baseline to 10.4% at the last visit, while the percentage of ALT values above ULN increased from 8% at baseline to 11.8% at the last visit. Considering each group separately, the baseline ALT and AST values were significantly higher in individuals with HIV-HBV/HBsAg-positive compared with cases with HIV mono-infection or cases with HIV-HBV/HBsAg loss (*p* < 0.001) and this difference mainly resided in the significantly higher AST and ALT values found at baseline in HBsAg-positive cases vs. HIV monoinfection. Over time, this difference gradually faded and significant difference was found between any groups at the annual visit corresponding to 60 months (Figures 1A,B).

The baseline prevalence of liver fibrosis in all patients was of 10.8% using APRI and of 9.9% using a Fib-4 score. At the visit corresponding to 60 months the prevalence of liver fibrosis in all patients was of 11.3% using the APRI score and of 10.8%, respectively using the Fib-4 score.

Considering each of the groups, the baseline prevalence of liver fibrosis using the APRI score was of 5.4% in patients with HIV mono-infection patients vs. 7.7% in individuals with HIV-HBV/HBsAg-negative and 16.8% in HIV-HBV/HBsAg-positive (*p*-value = 0.003). Conversely, the baseline prevalence of liver fibrosis using the Fib-4 score was of 7.2% in patients with HIV mono-infection vs. 20.5% in people with HBsAg loss and 21% in HBsAg-positive patients (*p* = 0.156). Over time, the difference between APRI and Fib-4 scores in these groups became non-significant. At the last visit corresponding to the 60 months-visit, the prevalence of liver fibrosis using the APRI score was of 8.1% in patients with HIV mono-infection vs. 12.8% in people with HBsAg loss and 16.1% in HBsAg-positive patients (*p* = 0.254). Similarly, the prevalence of liver fibrosis using a Fib-4 score reached 9% in patients with HIV mono-infection vs. 12.8% in in people with HBsAg loss and 12.9% in HBsAg-positive patients (*p* = 0.646).

Table 3 shows the prevalence of liver fibrosis in all patients as well as in each group, for the recorded annual assessments (at baseline, at 36 months, at 48 months, and at 60 months).

**Table 3:** The prevalence of liver fibrosis using APRI and Fib-4 score at baseline and at the subsequent annual evaluations corresponding to 36 months, 48 months, and 60 months in all patients and separately for individuals with HIV monoinfection, HIV-HBV co-infection with positive HBsAg and individuals with HIV-HBV and HBsAg loss.

**Patient group**	**Liver fibrosis prevalence**			
	**Baseline**	**Yearly assessment**	**Yearly assessment**	**Yearly assessment**
		**for 36 months**	**for 48 months**	**for 60 months**
**All patients (*n* = 212)**				
APRI >0.5 (%)	23 (10.8%)	27 (12.7%)	19 (9%)	24 (11.3%)
Fib-4 ≥1.45 (%)	21 (9.9%)	19 (9%)	20 (9.4%)	23 (10.8%)
**HIV mono-infection (*n* = 111)**				
APRI >0.5 (%)	6 (5.4%)	6 (5.4%)	4 (3.6%)	9 (8.1%)
Fib-4 ≥1.45 (%)	8 (7.2%)	8 (7.2%)	8 (7.2%)	10 (9%)
**HIV-HBV co-infection with positive HBsAg (*n* = 62)**				
APRI >0.5 (%)	14 (22.6%)	13 (21%)	9 (14.5%)	10 (16.1%)
Fib-4 ≥1.45 (%)	10 (16.1%)	6 (9.7%)	7 (11.3%)	8 (12.9%)
**HIV-HBV co-infection with HBsAg loss (*n* = 39)**				
APRI >0.5 (%)	3 (7.7%)	8 (20.5%)	6 (15.4%)	5 (12.8%)
Fib-4 ≥1.45 (%)	3 (7.7%)	5 (12.8%)	5 (12.8%)	5 (12.8%)

**Figure 2:**
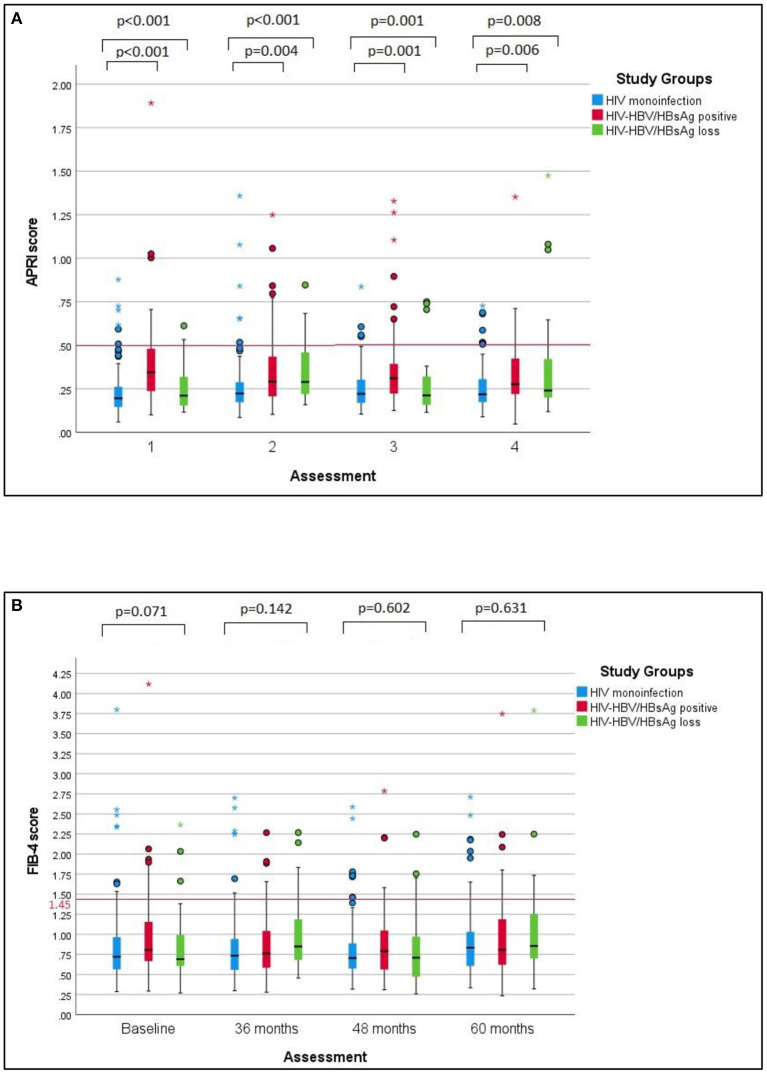
APRI and Fib-4 scores over time in the studied groups. **(A)** Comparative changes of APRI scores over time in the studied groups. APRI scores remained significantly higher in HIV-HBV/HBsAg-positive individuals versus individuals with HIV monoinfection or HIV-HBV/HBsAg loss throughout every visit. Additional pairwise comparisons revealed significantly higher median APRI scores between cases with positive HBsAg and cases with HIV monoinfection at every visit. **(B)** Comparative changes of Fib-4 sores over time in the studied groups. No significant differences were found between Fib-4 scores in any of the groups at baseline or at the subsequent visits corresponding to 36, 48 or 60 months.

All patients displayed an increase of APRI and Fib-4 scores between the first and final visit. In the case of patients with HIV mono-infection the median values of these scores displayed a statistically significant increase over the follow-up period (*p*-value = 0.009 for APRI and *p*-value = 0.013 for Fib-4). By comparison, no difference was found between the baseline and the final values of liver fibrosis scores irrespective of the presence or absence of HBsAg. The changes of liver fibrosis scores during follow-up period of 60 months are also represented in Figures 2A,B while the median values of APRI and Fib-4 values at baseline and at the final visit are displayed in Table 4.

**Table 4:** Median APRI and Fib-4 values at baseline and at the final visit corresponding to 60 months in all patients and separately for individuals with HIV mono-infection, HIV-HBV co-infection with positive HBsAg and individuals with HIV-HBV and HBsAg loss.

**Patient group**	**Baseline**	**Last visit**	***p*** **-values**
**All patients**			
Median APRI score (IQR)	0.220 (0.157,0.351)	0.241 (0.189, 0.351)	0.042
Median Fib-4 score (IQR)	0.753 (0.596, 1.045)	0.817 (0.614, 1.135)	0.005
**HIV mono-infection**			
Median APRI score (IQR)	0.195 (0.145, 0.263)	0.218 (0.172, 0.307)	0.009
Median Fib-4 score (IQR)	0.721 (0.561, 0.982)	0.833 (0.596, 1.039)	0.013
**HIV-HBV co-infection with positive HBsAg**			
Median APRI score (IQR)	0.299 (0.174, 0.425)	0.262 (0.202, 0.422)	0.705
Median Fib-4 score (IQR)	0.766 (0.636, 1.096)	0.816 (0.641, 1.201)	0.115
**HIV-HBV co-infection with HBsAg loss**			
Median APRI score (IQR)	0.218 (0.155, 0.340)	0.241 (0.198, 0.422)	1.000
Median Fib-4 score (IQR)	0.717 (0.621, 1.051)	0.855 (0.692, 1.261)	1.000

Only a few cases showed advanced liver fibrosis (APRI >1.5 or a Fib-4 score >3.25) during the study period.

At baseline, the prevalence of advanced liver fibrosis in cases with positive HBsAg was of 3.22% using an APRI >1.5 and of 1.6% using a Fib-4 score >3.25. No cases with negative HBsAg displayed advanced fibrosis according to these thresholds. Individuals with HIV mono-infection did not display any case of advanced fibrosis using an APRI >1.5. A Fib-4 score >3.25 was found in only 1 case (0.9%) with HIV mono-infection.

At the last visit, the prevalence of advanced liver fibrosis in patients with positive HBsAg remained at 3.22% using an APRI >1.5 and increased to 4.8% using a Fib-4 score >3.25. No individual with HBsAg loss showed an APRI >1.5, while the prevalence of advanced fibrosis using a Fib-4 score was of 5.1% in this group. At the last visit, no case with advanced liver fibrosis was recorded in patients with HIV mono-infection, using either APRI or Fib-4 scores.

Of note, 2 cases with HIV-HBV co-infection and positive HBsAg displayed both an APRI >1.5 and a Fib-4 >3.25 at the last visit. Both cases involved two young patients, aged 30 years at the last assessment, with an overall treatment duration exceeding 10 years, with fluctuating adherence to ART, low level HIV RNA (20 copies/ml) and CD4+ T cell counts >200 cells/mm^3^ (579 and 358 cells/mm^3^, respectively). Plasma HBV DNA was detectable in both cases (8,370,000 and 1,79,00,0000 IU/ml, respectively).

#### Risk factors for liver fibrosis in HIV and HIV/HBV patients

##### Risk factors associated with the presence of liver fibrosis over time

The predictors for the presence of liver fibrosis over the follow-up period were studied using a GEE model with logistic regression, as detailed in Table 5.

**Table 5:** Risk factors predicting the presence of liver fibrosis in all patients using APRI and Fib-4 scores over the study period (median duration of follow-up of 58.5 months). Liver fibrosis was defined using a APRI score >0.5 and for a Fib-4 score ≥1.45.

**Risk factors for liver fibrosis**	**Univariate analysis**		
	**OR**	**95% CI**	***P*** **-value**
**Presence of liver fibrosis using APRI >0.5**			
Current or previous HIV-HBV co-infection	4.245	2.132–8.452	<0.001
Male gender	0.393	0.210–0.737	0.004
CD4+T cell count (cells/mm^3^)	0.998	0.997–0.999	<0.001
CD4+T cell count <200 cells/mm^3^	3.512	1.381–8.830	0.002
CD4+ T cell count <500 cells/mm^3^	2.484	1.425–4.328	0.002
Log10 plasma HIV RNA	1.372	1.030–1.829	0.018
Detectable HIV RNA	1.760	1.047–2.958	0.022
Serum cholesterol (mg/dl)	0.984	0.973–0.994	0.002
Serum triglycerides (mg/dl)	0.994	0.989–0.998	0.003
**Presence of liver fibrosis using Fib-4 ≥1.45**			
Male gender	2.760	1.202–6.336	0.017
CD4+ T cell count	0.998	0.997–0.999	<0.001
CD4+ T cell count <200 cells/mm^3^	2.414	1.014–5.747	0.016
CD4+ T cell count <500 cells/mm^3^	2.836	1.501–5.355	0.001
Log10 plasma HIV RNA	1.407	1.031–1.919	0.005
Serum cholesterol (mg/dl)	0.988	0.978–0.998	0.003
Non-adherence to ART	2.641	1.070–6.515	0.035

The presence of an APRI score >0.5 over time was independently associated with the HIV-HBV co-infection, irrespective on the presence of HBsAg (*p* < 0.001), male gender (*p* = *0.004*), log10 plasma HIV RNA (*p* = 0.018), detectable plasma HIV RNA (*p* = 0.022), CD4+T cell count <200 cells/mm^3^ (*p*< *0.002*), as well as with a CD4+T cell count <500 cells/mm^3^ (*p* = 0.005).

By comparison, the presence of liver fibrosis as indicated by a Fib-4 score ≥1.45 over the follow-up period was independently associated with the male gender (*p* = *0.013*), CD4+T cell counts values (*p*< *0.001*) and specific CD4+T cell count thresholds <200 cells/mm^3^ (*p* = *0.046)* or <500 cells/mm^3^ (*p* = 0.001). Non-adherence to ART over the study period was also associated with the presence of a Fib-4 score ≥1.45 (*p* = 0.035).

##### Risk factors for the presence of liver fibrosis on the last visit

The risk factors for liver fibrosis were reassessed on the last visit to verify which factors continue to remain associated with liver fibrosis under prolonged ART.

##### All patients

The risk factors independently associated with liver fibrosis in the overall population at the annual visit corresponding to 60 months are represented in Table 6.

**Table 6:** Risk factors for the presence of liver fibrosis at the last visit, corresponding to the visit at 60 months, using chi-square tests and non-parametric Mann Whitney U-tests.

**Risk factors for liver fibrosis**	**Liver fibrosis (APRI** >**0.5)**	***p*** **-value**	**Liver fibrosis (Fib-4** ≥**1.45)**	***p*** **-value**
HIV-HBV/positive HBsAg	10 (16.7%)	0.254	8 (13.3%)	0.646
HIV-HBV/negative HBsAg	5 (13.2%)		5 (12.8%)	
HIV monoinfection	9 (8.3%)		10 (9.1%)	
Male gender (%)	8 (33.3%)	0.030	16 (69.6%)	0.103
Median CD4 T cell count, IQR (cells/mm^3^)	512 (226, 623)	<0.001	552 (369, 676)	0.010
CD4 T cells <200 cells/mm^3^ (%)	3 (13.6%)	0.010	1 (4.5%)	0.445
CD4 T cells <500 cells/mm^3^	10 (45.5%)	0.023	9 (40.9%)	0.088
Log10 HIV RNA (copies/ml)	1.65 (1.33, 4.6)	0.119	1.51 (1.32, 3.11)	0.675
Detectable Plasma HIV RNA	10 (50%)	0.261	9 (50%)	0.306
Serum cholesterol (mg/dl)	180 (137, 200)	0.020	178 (146, 220)	0.039
Serum tryglicerides (mg/dl)	108 (72, 139)	0.018	121 (94, 200)	0.655
Non-Adherence to ART (%)	6 (31.6%)	0.006	6 (28.6%)	0.019

While the only cases with advanced fibrosis at the final visit were recorded in the group with positive HBsAg, no statistically significant difference was found between the presence of liver fibrosis in this group and patients with negative HBsAg or HIV monoinfection using either an APRI score >0.5 (*p* = 0.254) or a Fib-4 score ≥1.45 (*p* = 0.646).

Risk factors for liver fibrosis included a CD4 T cell count <200 cells/mm^3^ or <500 cells/mm^3^ for the APRI score, while both APRI and Fib-4 scores were associated with a lower level of cholesterol (possibly due to the development of liver disease) and non-adherence to ART.

In particular, non-adherence to ART significantly increased the risk of liver fibrosis as defined by APRI >0.5 by 4.21 folds (OR =4.212, 95%CI [1.407, 12.607], *p* = 0.006), while the risk of liver fibrosis as defined by a Fib-4 score≥1.45 increased by 3.41 folds (OR = 3.412, 95%CI [1.168, 9.965], *p* = 0.019).

Notably, the wider use of INSTIs, with a potentially favorable effect on the development of liver steatosis (31, 32), was not associated with the concentrations of serum cholesterol or triglycerides (*p* = 0.655 and *p* = 0.282, respectively). Furthermore, the use of INSTIs was not associated with the presence of liver fibrosis as defined using an APRI >0.5 (*p* = 0.292) or using Fib-4 score ≥1.45 (*p* = 0.545). Similarly, the use of PIs with a negative impact on lipid profile (32) was not associated with liver fibrosis using either APRI >0.5 (*p* = 0.103) or Fib-4 score (*p* = 0.103).

##### Patients with HIV mono-infection

We found no association between liver fibrosis and immunologic, virologic or metabolic parameters in this group of individuals with HIV monoinfection at the last visit, corresponding to the assessment corresponding to 60 months.

##### Patients with HIV-HBV co-infection and positive HBsAg

Median CD4 T cell counts were significantly lower in cases with positive HBsAg and with an APRI score >0.5 on the last visit (358 vs. 638 cells/mm^3^, *p*-value = 0.002). However, this finding was not observed for the Fib-4 score (*p* = 0.124). No other correlations were found between other immunologic or metabolic parameters and liver fibrosis.

The presence of a detectable serum HBV DNA on the final visit was not associated with liver fibrosis using an APRI score (*p* = 0.056) or a Fib-4 score (*p* = 0.205). Similarly, the values of plasma HBV DNA were not associated with an APRI >0.5 (*p* = 0.085) or with a Fib-4 score ≥1.45 (*p* = 0.283).

##### Patients with HIV-HBV co-infection and HBsAg loss

No association was found between immunologic or metabolic parameters and the presence of liver fibrosis in this group of patients.

## Discussion

The assessment of liver disease remains an important issue in PLWH with prolonged ART to ensure a timely detection and follow-up of liver-related complications. Non-invasive methods have been increasingly used to document the prevalence of liver fibrosis in HIV and HIV-HBV infected patients (33–35), yet data on the progression or regression of liver fibrosis while on ART are scarce (22, 34, 36–38).

The current study offers a comparative perspective on the longitudinal changes of non-invasive APRI and Fib-4 scores in a population of young HIV patients with a median ART exposure of 10.5 years, including individuals with HBV co-infection and positive HBsAg or HBsAg seroclearence. Thus, at the last annual visit corresponding to 60 months, the APRI score in all patients increased with 0.5% reaching a liver fibrosis prevalence of 11.3%, while the Fib-4 score increased with 0.9%, reaching a liver fibrosis prevalence of 10.8%.

Individuals with current HIV-HBV co-infection showed higher APRI and Fib-4 values compared with cases with HIV mono-infection on long-term treatment. Furthermore, the study showed that individuals with both HIV-HBV co-infection and HBsAg loss are more likely to display liver fibrosis scores indicative of liver fibrosis compared with individuals with HIV mono-infection. The high prevalence of liver fibrosis using both non-invasive scores and imaging data has been also reported in other studies on HIV-HBV patients with positive HBsAg even under ART Hence, the prevalence of liver fibrosis in cases with HIV-HBV co-infection has been shown to vary between 12.3 and 33% (4, 17, 18, 25, 36), also reaching rates of 37% (39) or 43% (40) in certain studies. Data on the progression or regression of liver fibrosis in cases with HBsAg seroconversion is limited. In a study by Dezanet et al. which included 12 individuals with HIV-HBV co-infection and HBsAg seroclearence, 10 of these patients continued to display moderate fibrosis/cirrhosis over a period of 7.6 years of ART with TDF and the cases with HBsAg seroclearence and liver cirrhosis represented 28.6% of all cases with cirrhosis (10). An additional aspect related to cases with HBsAg loss regards the diagnosis and follow-up of occult hepatitis B. In this respect, a certain subset of patients with HIV and isolated anti-HbcIgG Ab develop ongoing low-level replication of HBV DNA and are classified as occult hepatitis B. The latter is characterized by persistent plasma and intrahepatic replication, which in turn leads to liver inflammation and fibrosis as well as to an increased risk of hepatocellular carcinoma (41–43). Hence, these individuals continue to require a careful surveillance of liver disease even after HBsAg loss.

In our study, the prevalence of liver fibrosis at the last visit in individuals with HIV mono-infection was of 8.1% using the APRI score and of 9% using the Fib-4 score, comparable with data from studies using non-invasive methods (20, 23, 33, 44). Additionally, the current study showed a significant increase of APRI and Fib-4 scores over time in this group with high CD4 T cell counts and relatively good real-life adherence to ART.

Regarding the risk factors for liver fibrosis, it is expected that the cumulative exposure to ART leads to a gradual decline on the impact of various risk factors for liver fibrosis, In this respect, studies on patients undergoing ART show discordant data on various risk factors for liver fibrosis such as advancing age, male gender, detectable HIV RNA, lower CD4 T cell counts as well as the presence of HBV/HCV co-infection and detectable plasma HBV DNA (4, 16, 17, 33, 36, 39). Individuals with HIV-HBV co-infection and well-controlled viral loads on prolonged ART display lower scores of liver fibrosis, comparable with the scores found in people with HIV mono-infection (36) and a lower rate of significant liver-related complications (8, 22). Nevertheless, these patients are less likely to display a regression of liver fibrosis despite the ongoing administration of ART. Additionally, distinct cases of PLWH with a good virologic and immunologic control under ART can still progress to liver fibrosis (11, 17, 20). One hypothesis explaining this progression resides in the contribution of metabolic factors over time. Fatty liver disease and steatohepatitis are found in one third of HIV-HBV patients with undetectable viral loads (39, 45) and in 30–65% of PLWH (46). On the long-term, the progression of steatosis is associated with an increased severity of liver fibrosis. In this regard, we observed significantly higher baseline values of serum cholesterol in cases with HIV mono-infection vs. cases with HIV-HBV co-infection and either positive HBsAg or HBsAg loss. While these values decreased by the last visit, additional data is needed to verify whether this decrease was related to the development of liver failure and to understand the alterations of the metabolic pathways under ART. Regarding the potential role of ART as a cumulative risk factor for the development of liver fibrosis, we did not find a correlation between anti-re-trovirals and APRI or Fib-4 scores, in agreement with other authors (33).

Serum-based scores for the surveillance of liver fibrosis have been suggested as screening tools and are easy to access and more affordable, especially in low-income settings. Nevertheless, the use of such scores demands an optimization of liver fibrosis thresholds in populations on long-term ART. In our study, approximately only 10% of all patients showed ALT and AST values above the upper limit of the assay. Additionally, the median values of APRI and Fib-4 scores remained well-below the threshold for liver fibrosis in the overall population and in each subgroup. This finding is in agreement with the values obtained in longitudinal studies by Sterling et al. (34) and Yang et al. (33). Therefore, individuals with HIV-HBV co-infection are more likely to display lower scores of liver fibrosis while on ART (20, 36, 39, 47), similar to patients with chronic hepatitis B on antiviral treatment (48–51). The current study also showed low scores of liver fibrosis in cases with HIV monoinfection, as found in other studies on PLWH on ART (16, 17, 22, 25, 36, 38, 52). Hence, it is probable that similar to patients with chronic hepatitis B (53), distinct lower thresholds liver fibrosis are needed in PLWH on extensive ART. Sterling et al. (34) have indicated lower optimal cut-offs for both APRI and Fib-4 scores in treated patients, while other authors have underlined the discordant results obtained using traditional APRI and Fib-4 cut-offs in PLWH without elevated transaminases (44). Nevertheless, considering the increase of the scores found over time in the cases with HIV mono-infection, a rigorous follow-up using at least serum-based scores should be considered in all PLWH on prolonged ART, irrespective on the presence of elevated transaminases or of other viral co-infections.

The study has certain limitations including its retrospective approach and the use of serum-based models. Secondly, a complete monthly pill release was not available for 10 patients and records on HBV-DNA were not available in all patients. A more detailed assessment of the lipid metabolism, liver steatosis and metabolic syndrome could have helped characterize its importance for the development of liver fibrosis.

Still, the current study remains one of the few longitudinal studies documenting the variations of APRI and Fib-4 scores in cases with HIV and HIV-HBV co-infection including cases with HBsAg loss. Moreover, the study follows a group of individuals with HIV mono-infection less studied, namely young patients horizontally infected with HIV during early childhood and with prolonged ART exposure.

In conclusion, the study documents the discrete increase of liver fibrosis scores in individuals with long-term ART, including patients with HIV mono-infection. Additionally, the presence of liver fibrosis in all patients throughout a median period of 58.5 months was associated with higher plasma log10 HIV RNA, lower CD4 T cell counts, HIV-HBV co-infection irrespective on the presence/absence of HBsAg (for the APRI score) and non-adherence to ART (for the Fib-4 score). The study also underlines the changes in the risk factors for liver fibrosis over time, due to ART, given that at the last visit the CD4 T cell count and non-adherence to ART were still associated with liver fibrosis.

All in all, APRI and Fib-4 scores offer an inexpensive and accessible alternative for a careful follow-up of liver fibrosis in all PLWH, including cases with HIV-HBV co-infection and HBs loss or HIV mono-infection without elevated transaminases who are less likely to be referred for other liver fibrosis assessments.

## Data availability statement

The raw data supporting the conclusions of this article will be made available by the authors, without undue reservation.

## Ethics statement

The studies involving human participants were reviewed and approved by Ethics Committee of Prof. Dr. Matei Bals National Institute of Infectious Diseases. The patients/participants provided their written informed consent to participate in this study.

## Author contributions

DI: conception and design. DI, ML, OB, A-MT, and CO: collection and assembly of data. DI: data analysis and interpretation. SR and SI: critial revision. All authors has approved the submitted version.

## Conflict of interest

The authors declare that the research was conducted in the absence of any commercial or financial relationships that could be construed as a potential conflict of interest.

## Publisher's note

All claims expressed in this article are solely those of the authors and do not necessarily represent those of their affiliated organizations, or those of the publisher, the editors and the reviewers. Any product that may be evaluated in this article, or claim that may be made by its manufacturer, is not guaranteed or endorsed by the publisher.
